# Identifying Tumorigenesis and Prognosis-Related Genes of Lung Adenocarcinoma: Based on Weighted Gene Coexpression Network Analysis

**DOI:** 10.1155/2020/4169691

**Published:** 2020-02-28

**Authors:** Ming Yi, Tianye Li, Shuang Qin, Shengnan Yu, Qian Chu, Anping Li, Kongming Wu

**Affiliations:** ^1^Department of Oncology, Tongji Hospital of Tongji Medical College, Huazhong University of Science and Technology, Wuhan 430030, China; ^2^Department of Obstetrics and Gynecology, Tongji Hospital, Tongji Medical College, Huazhong University of Science and Technology, Wuhan, Hubei 430030, China; ^3^Department of Medical Oncology, The Affiliated Cancer Hospital of Zhengzhou University and Henan Cancer Hospital, Zhengzhou, China

## Abstract

Lung adenocarcinoma is the most frequently diagnosed subtype of nonsmall cell lung cancer. The molecular mechanisms of the initiation and progression of lung adenocarcinoma remain to be further determined. This study aimed to screen genes related to the progression of lung adenocarcinoma. By weighted gene coexpression network analysis (WGCNA), we constructed a free-scale gene coexpression network to evaluate the correlations between multiple gene sets and patients' clinical traits, then further identify predictive biomarkers. GSE11969 was obtained from the Gene Expression Omnibus (GEO) database which contained the gene expression data of 90 lung adenocarcinoma patients. Data of the Cancer Genome Atlas (TCGA) were employed as the validation cohort. After the average linkage hierarchical clustering, a total of 9 modules were generated. In the clinical significant module (*R* = 0.44, *P* < 0.0001), we identified 29 network hub genes. Subsequent verification in the TCGA database showed that 11 hub genes (*ANLN*, *CDCA5*, *FLJ21924*, *LMNB1*, *MAD2L1*, *RACGAP1*, *RFC4*, *SNRPD1*, *TOP2A*, *TTK*, and *ZWINT*) were significantly associated with poor survival data of lung adenocarcinomas. Besides, the results of receiver operating characteristic curves indicated that the mRNA levels of this group of genes exhibited high specificity and sensitivity to distinguish malignant lesions from nonmalignant tissues. Apart from mRNA levels, we found that the protein abundances of these 11 genes were remarkably upregulated in lung adenocarcinomas compared with normal tissues. In conclusion, by the WGCNA method, a panel of 11 genes were identified as predictive biomarkers for tumorigenesis and poor prognosis of lung adenocarcinomas.

## 1. Introduction

Lung cancer is the leading cause of cancer-related deaths all over the world and more than 80% of lung cancers are diagnosed as nonsmall cell lung cancers (NSCLCs) [1, 2]. As the most common subtype of NSCLC, the incidence of lung adenocarcinoma (LUAD) is increasing year by year [3]. Historically, the standard care for advanced LUAD was cytotoxic chemotherapy-involved comprehensive treatment. Due to the deeper understanding of genomics and tumorigenesis-associated molecular pathways, molecularly targeted therapies have been developed and a number of LUAD patients with these specific gene alterations could benefit from these regimens [4].

A growing body of evidence indicates that although gene alterations accumulate during the development of LUADs, a proportion of LUADs are primarily driven by single gene alterations such as epidermal growth factor receptor (*EGFR*) mutation and (anaplastic lymphoma kinase) *ALK* or *ROS1* rearrangement, which are also known as driven genes [5–8]. As the most frequent oncogenic driver, nearly 10%–15% population harbors *EGFR* mutation in patients with LUAD especially young nonsmokers [9]. Drugs targeting driven genes show a more potent anticancer effect and lower toxicity compared with conventional chemotherapies; thus multiple molecular targeted agents have been approved by the U.S. Food and Drug Administration for LUAD treatment [10]. The results of phase III clinical trial IPASS strongly support using gefitinib as first line treatment for advanced EGFR mutation-driving LUAD patients [11]. However, in spite of the increasing amount of confirmed targetable oncogenic drivers (including but not limited to *EGFR*, *ALK*, *ROS1*, *RET*, *BRAF*, *HER2*, *MET*, *KRAS*, and *NTRK*), there are about half of LUADs without known driven genes and treatment targets [12, 13]. Therefore, it is meaningful to investigate the molecular mechanisms associated with the initiation and progression of LUAD. Screening more candidate genes might be helpful to molecular diagnosis and the development of targeted agents.

The weighted gene coexpression network analysis (WGCNA) is a widely utilized technique to generate free-scale coexpression network which contributes to screen the modules containing highly correlated genes [14]. By analyzing large-scale gene expression data sets with patients' clinicopathological parameters, WGCNA could be utilized to identify potential treatment targets and predictive biomarkers [15]. In this study, we described gene coexpression patterns via a WGCNA-based systematic biology analysis method and identified a panel of biomarkers associated with the tumorigenesis and outcomes of LUADs.

## 2. Materials and Methods

### 2.1. Data Procession

**Figure 1:**
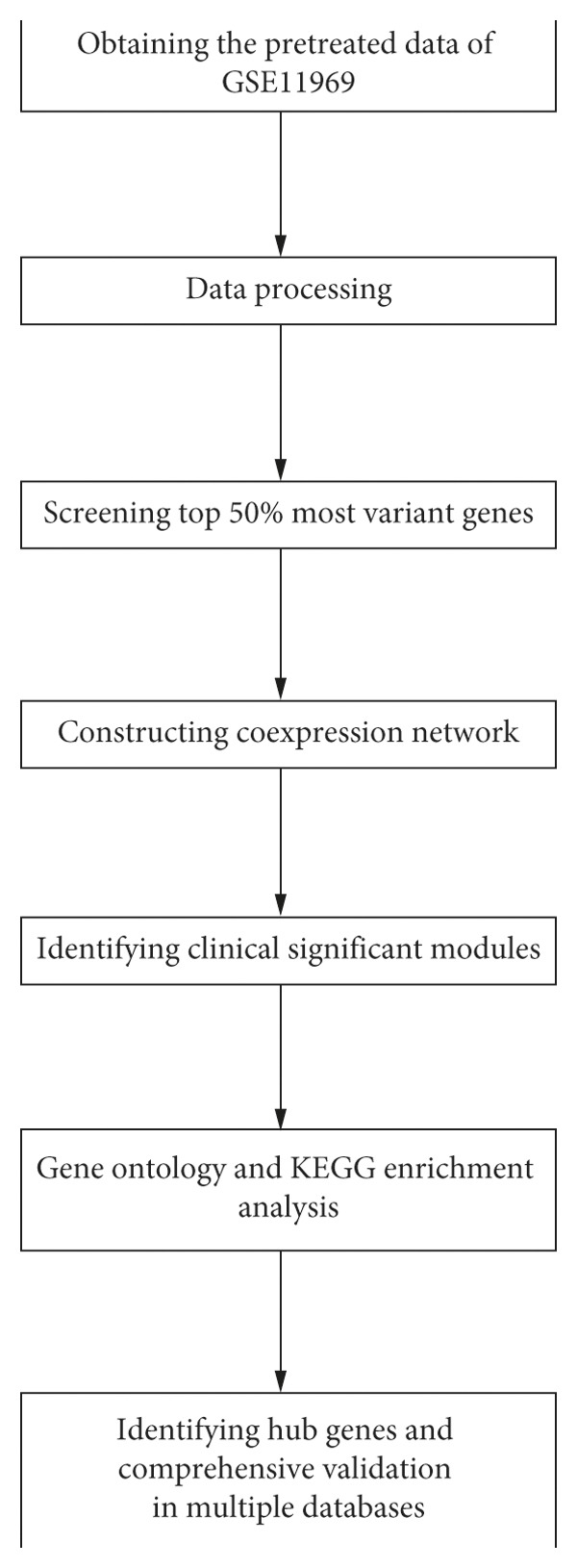
The flow chart of this study.

This study was conducted following workflow including data acquisition, WGCNA network construction, and hub genes identification (Figure 1). The gene expression matrix (GSE11969) was downloaded from the Gene Expression Omnibus (GEO) database (https://www.ncbi.nlm.nih.gov/geo/query/acc.cgi?acc=GSE11969) [16]. GSE11969 contains the gene expression values of 90 LUAD patients based on platform GPL7015 (Agilent *Homo sapiens* 21.6K custom array). After LOWESS normalized, background subtracted, the expression value data were calculated as log10 of processed Red signal/processed Green signal. We utilized pretreated data and selected the top 50% variant genes (8092 genes) via variance analysis for further WGCNA.

### 2.2. Coexpression Network Construction

By R software (version 3.6.0) with the WGCNA package, the gene coexpression network was constructed based on the expression data of 8092 genes [14]. We performed the analysis as previously described [14]. We introduced intermediate quantity coexpression similarity *S*_*ij*_ to reflect the connection strength between genes as in the following formula:

(1) $$\begin{array}{c} S_{ij}=\left|\text{cor}\left(x_{i},x_{j}\right)\right|,\\ a_{ij}=S_{ij}^{\beta }. \end{array}$$

The aforementioned *x*_*i*_ and *x*_*j*_ are the vectors of the expression values of two different genes *i* and *j*. Cor represents the Pearson correlation coefficient of the two vectors. This transition aims to increase the weight of strong connections and decrease the weight of weak connections [17]. In this study, *β* = 3 (scale-free *R*^2^ > 0.90) was adopted as a soft-thresholding index to construct a scale-free coexpression network. In this coexpression network, genes with strong connections would be clustered into one module. Based on adjacency matrix, we calculated topological overlap measure (TOM) which is the surrogate measuring the network connectivity of a certain gene by summing its adjacency of all other components of the network. Then, we created a hierarchical clustering tree. Under the condition of setting minimum cluster size as 50 and height as 0.25, 9 modules were generated via Dynamic Tree Cut algorithm.

### 2.3. Screening Clinical Significant Modules

The correlations between clustered modules and patients' traits were estimated by module eigengenes (MEs) and module gene significance (MS). MEs referred to the first principal component in each single module. The values of MEs could serve as the surrogate of the expression levels of all genes in the module. Thus, clinical significant modules could be identified by calculating the correlations between MEs and clinic-pathological parameters. Besides, gene significance (GS) referred to the *P* value (calculated in logs as lgP-value) of the linear regression analysis between gene expressions and samples' characteristics. The MS means the average GS of all genes in one module.

### 2.4. Gene Ontology Terms and KEGG Pathways Enrichment Analysis

G:Profiler (https://biit.cs.ut.ee/gprofiler) is an online analysis tool for functional enrichment which contains the data of Ensembl database, Gene Ontology (GO) terms, Kyoto Encyclopedia of Genes and Genomes (KEGG) pathway, Reactome, and WikiPathways et al. GO terms, and KEGG pathways enrichment analyses were performed by g:Profiler (version: e95_eg42_p13_f6e58b9). The GO terms consisted of three categories: biological process (BP), cellular component (CC), and molecular function (MF). With the cut-off value as a false positive rate (FDR) < 0.05, the significantly enriched GO terms as well as KEGG pathways were screened out.

### 2.5. Identifying Hub Genes

Hub genes were defined as genes possessing high connectivity with other genes in the same module (the absolute value of cor.geneModuleMembership ≥ 0.8). Besides, hub genes of modules with clinical significance were prone to highly correlate with corresponding clinical traits (the absolute value of cor.geneTraitSignificance ≥ 0.2). To further confirm the identified hub genes, we utilized the online tool Kaplan–Meier Plotter (http://kmplot.com/analysis/) for prognostic analysis in LUAD populations [18]. Besides, we downloaded pretreated LUAD expression data from the TCGA database (https://xenabrowser.net/) and analyzed the correlation between the hub gene expression and clinical parameters. Apart from the mRNA level, we employed the Human Protein Atlas database (http://www.proteinatlas.org) to confirm the role of hub genes in tumorigenesis in protein abundances.

## 3. Results

### 3.1. Constructing Weighted Coexpression Network and Identifying Clinical Significant Module

**Figure 2:**
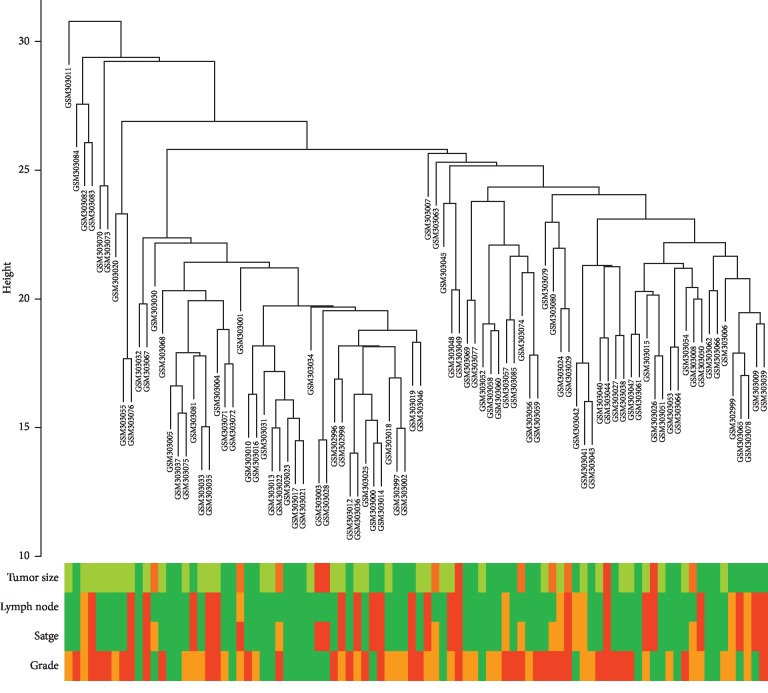
Clustering dendrogram of 90 LUAD samples.

**Figure 3:**
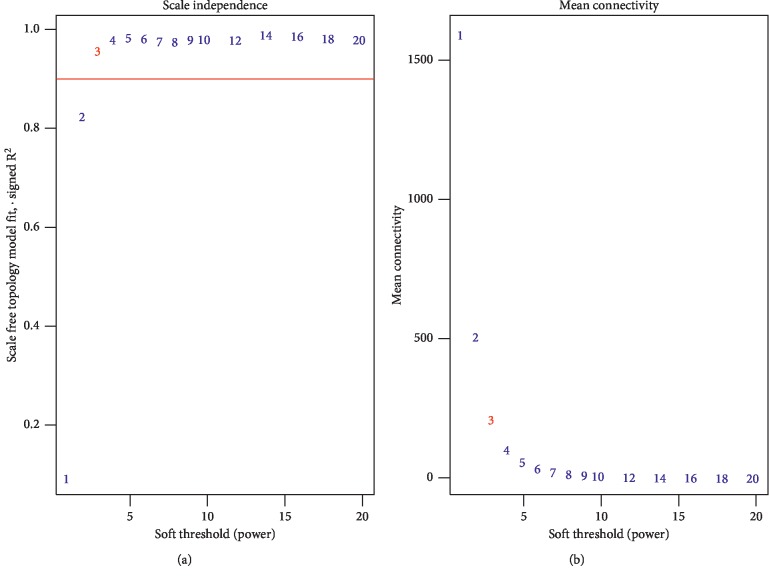
Determining the soft-thresholding power. (a) Analyzing the scale-free fit index under the background of different soft-thresholding powers (*β*). (b) Analyzing mean connectivity when using different soft-thresholding powers.

**Figure 4:**
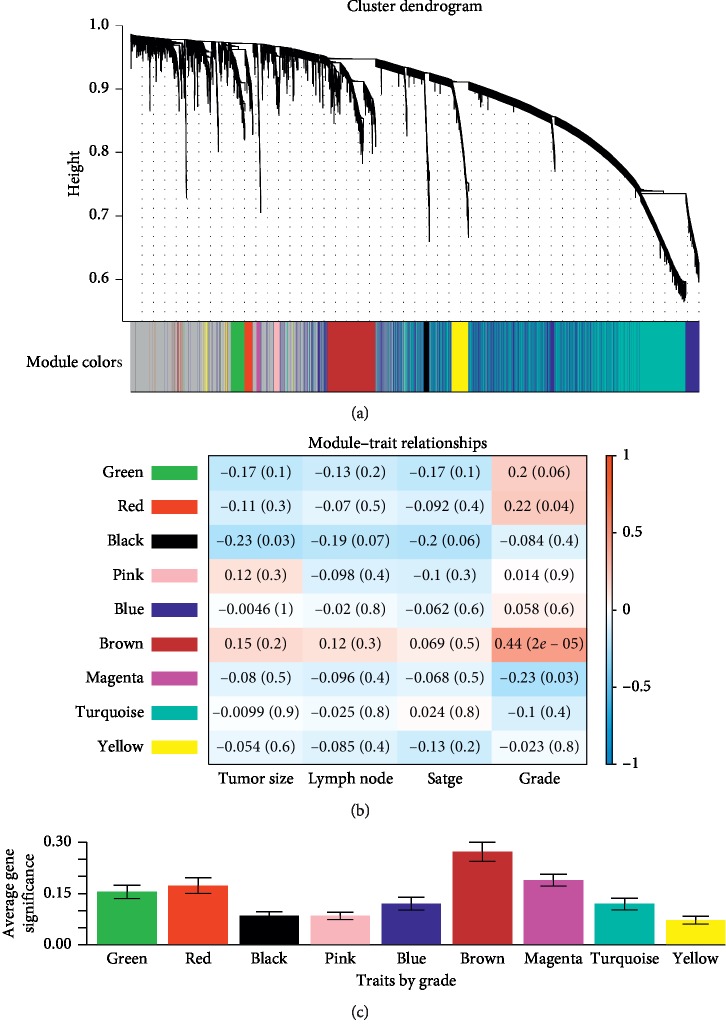
Identifying LUAD-associated clinical significant modules. (a) The dendrogram of top 50% most variant genes which were clustered by the Dynamic Tree Cut algorithm. (b) The heat map describing the correlation between all module eigengenes and clinical traits including tumor size, lymph node metastasis, TNM stage, and tumor differentiation grade. (c) The histogram describing the relationship between the average gene significance of all modules and tumor differentiation grade.

The total 90 LUAD samples were clustered by Pearson's correlation and average linkage algorithms (Figure 2). Then, we conducted coexpression analysis. In this study, the soft-thresholding power was set to *β* = 3 (*R*^2^ > 0.90) to generate a scale-free gene coexpression network (Figure 3). Eventually, 9 modules were generated by average linkage hierarchical clustering. The brown module had the highest correlation with tumor differentiation (*R* = 0.44, *P* < 0.0001) (Figure 4). Therefore, the brown module was identified as the one with clinical significance, which was used for the following analysis.

### 3.2. GO Terms and KEGG Pathway Enrichment

**Figure 5:**
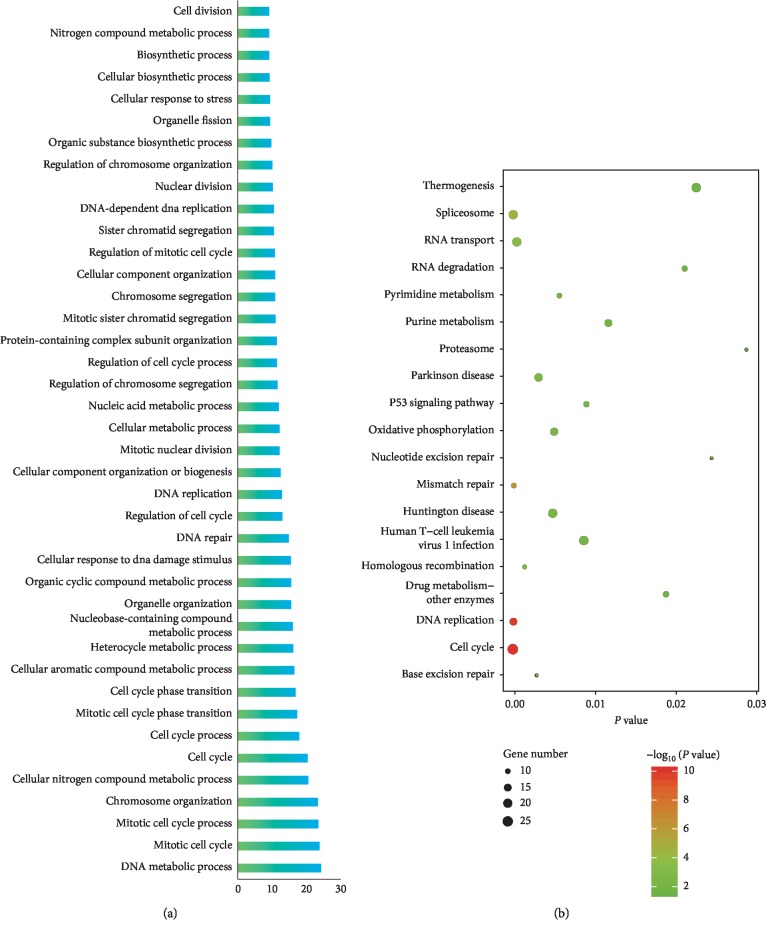
GO terms and KEGG pathway enrichment analysis of genes in the brown module. (a) GO-biological process enrichment analysis. (b) KEGG pathway enrichment analysis.

**Figure 6:**
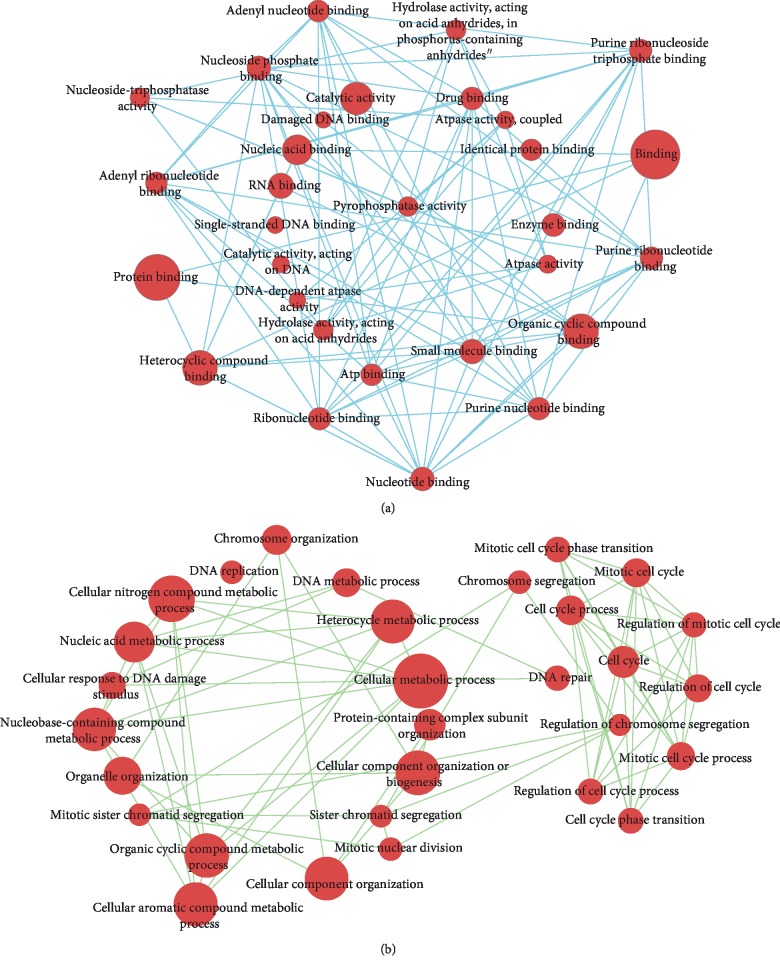
Interaction networks between enriched GO-biological process and cellular component terms. (a) Enriched GO-biological process terms based interaction networks. (b) Enriched GO-cellular component terms based interaction networks.

**Figure 7:**
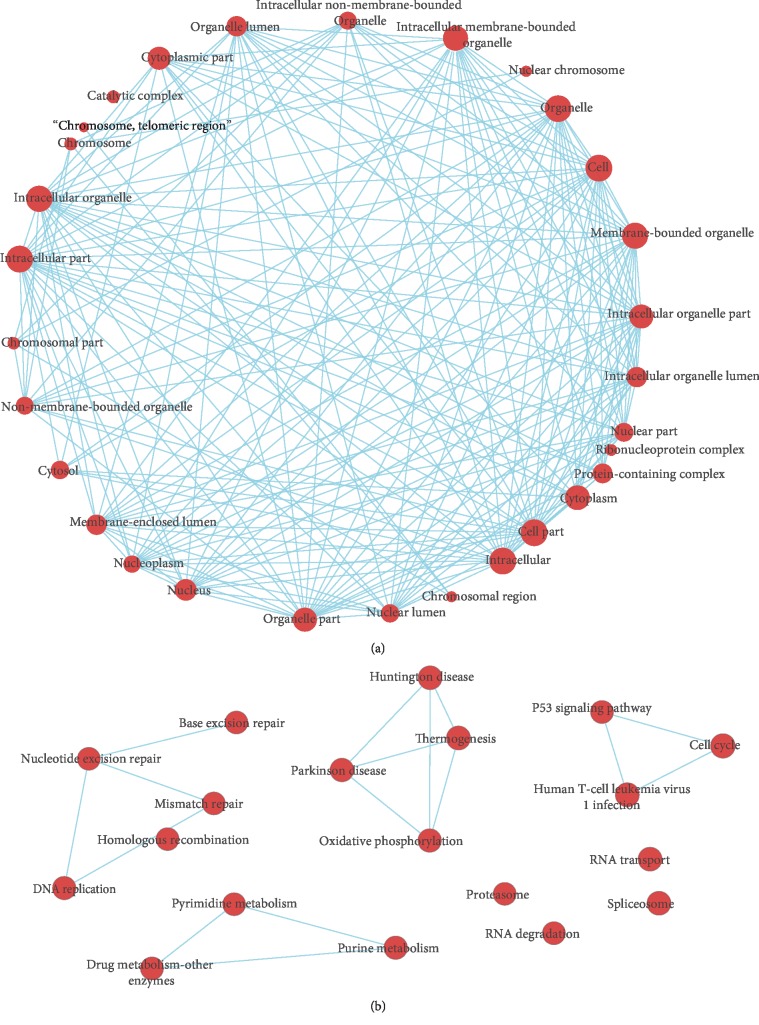
Interaction networks between enriched GO-molecular function terms and KEGG pathways. (a) Enriched GO-molecular function terms based interaction networks. (b) Enriched KEGG pathways based interaction networks.

To get an overall understanding of 725 genes in the brown module, we conducted GO terms and KEGG pathway enrichment. The results of GO-BP terms and KEGG pathway enrichment showed that genes within brown modules were significantly enriched in cell cycle-associated processes (such as “mitotic cell cycle,” “mitotic cell cycle process,” “cell cycle,” “mitotic cell cycle phase transition,” “cell cycle phase transition,” and “regulation of cell cycle”) as well as DNA damage repair-related processes (including “cellular response to DNA damage stimulus,” “DNA repair,” “mismatch repair,” “nucleotide excision repair,” and “base excision repair”) (Figure 5). Besides, a significant enrichment in multiple cancer-related pathways such as “p53 signaling pathway” and “human T−cell leukemia virus 1 infection” was observed. Additionally, by Cytoscape software (version 3.6.0), we constructed interaction networks between the enriched GO terms and KEGG pathways (Figures 6 and 7).

### 3.3. Hub Genes Identification and Comprehensive Validation in Multiple Database

**Figure 8:**
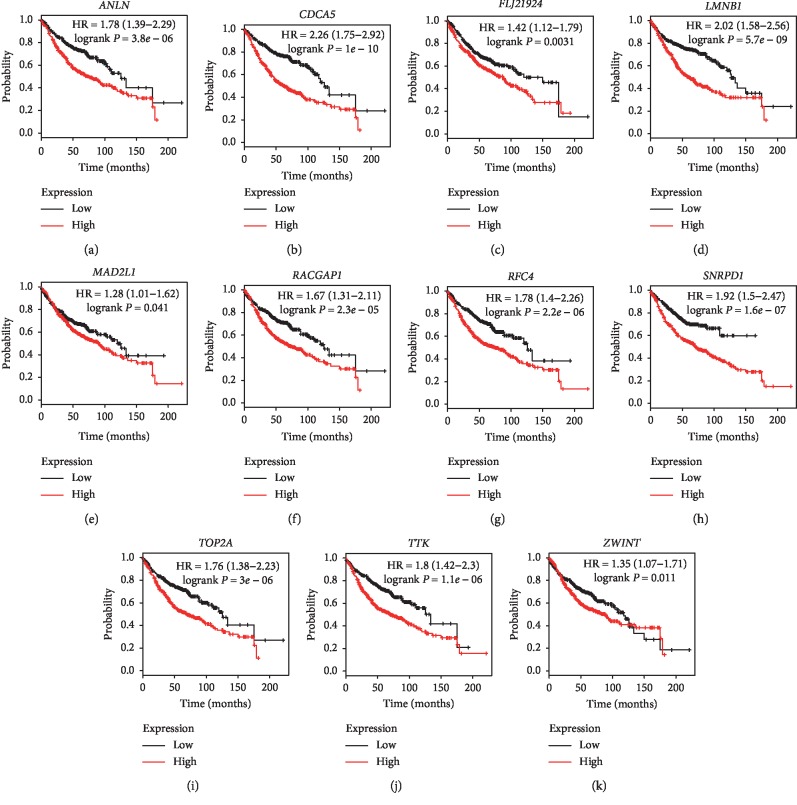
Overall survival analysis of the 11 hub genes in LUADs by online tool Kaplan–Meier plotter. The patients were classified into high-expression group and low-expression group based on the median mRNA levels of (a) *ANLN*, (b) *CDCA5*, (c) *FLJ21924*, (d) *LMNB1*, (e) *MAD2L1*, (f) *RACGAP1*, (g) *RFC4*, (h) *SNRPD1*, (i) *TOP2A*, (j) *TTK*, and (k) *ZWINT*.

**Figure 9:**
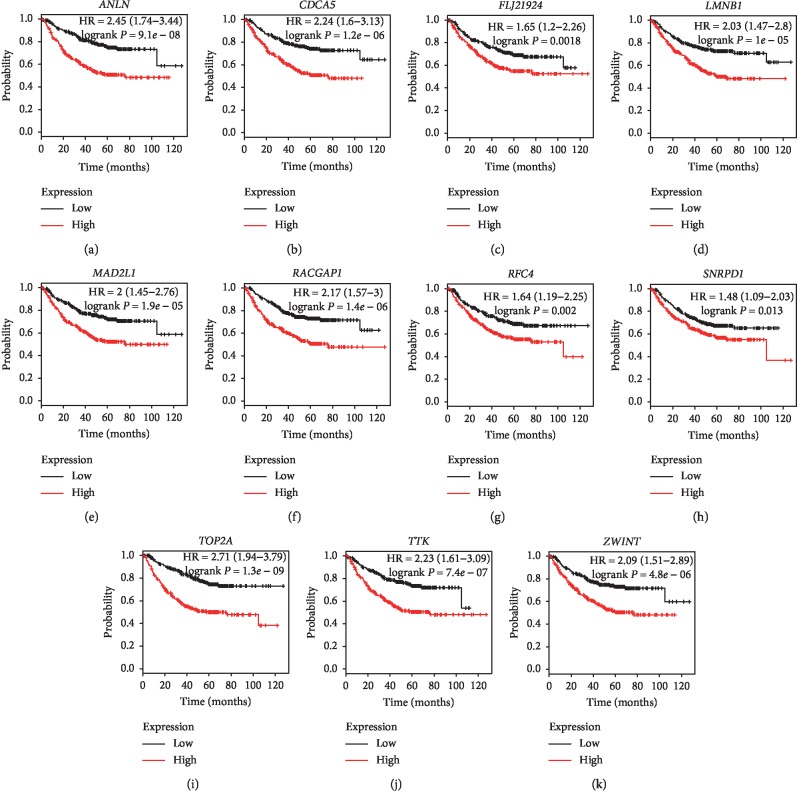
Progression-free survival analysis of the 11 hub genes in LUADs by online tool Kaplan–Meier plotter. The patients were classified into high-expression group and low-expression group based on the median mRNA level of (a) *ANLN*, (b) *CDCA5*, (c) *FLJ21924*, (d) *LMNB1*, (e) *MAD2L1*, (f) *RACGAP1*, (g) *RFC4*, (h) *SNRPD1*, (i) *TOP2A*, (j) *TTK*, and (k) *ZWINT*.

**Figure 10:**
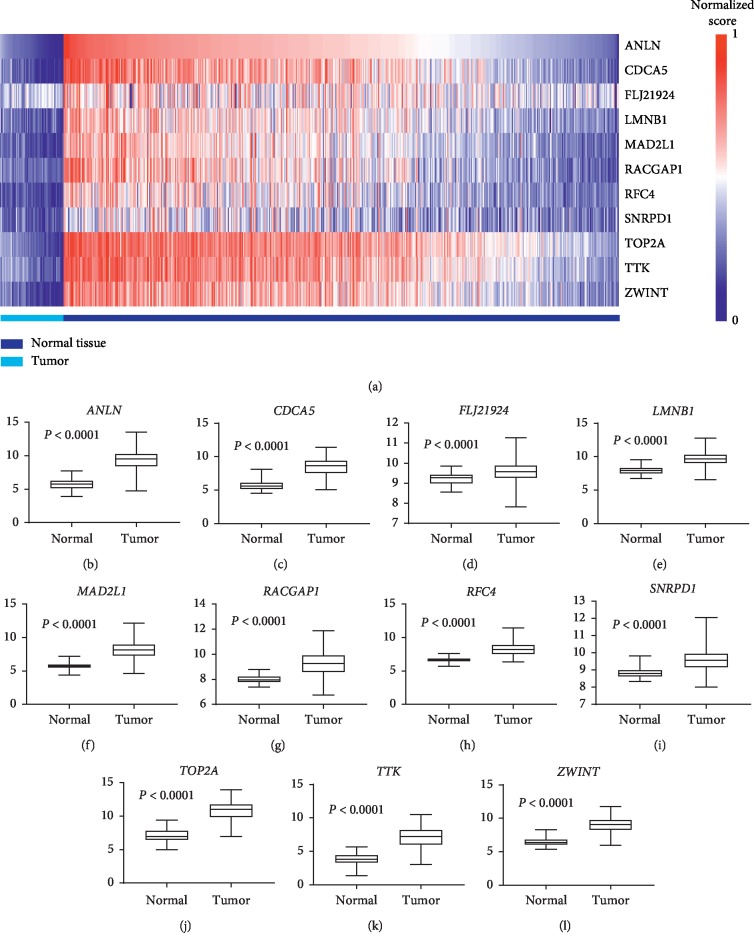
Gene expression levels of the 11 hub genes in normal tissues and LUADs. (a) The heat map of mRNA levels of *ANLN*, *CDCA5*, *FLJ21924*, *LMNB1*, *MAD2L1*, *RACGAP1*, *RFC4*, *SNRPD1*, *TOP2A*, *TTK*, and *ZWINT* in primary tumors and normal tissues. The comparisons of mRNA levels of (b) *ANLN*, (c) *CDCA5*, (d) *FLJ21924*, (e) *LMNB1*, (f) *MAD2L1*, (g) *RACGAP1*, (h) *RFC4*, (i) *SNRPD1*, (j) *TOP2A*, (k) *TTK*, and (l) *ZWINT* between primary tumors and normal tissues.

**Figure 11:**
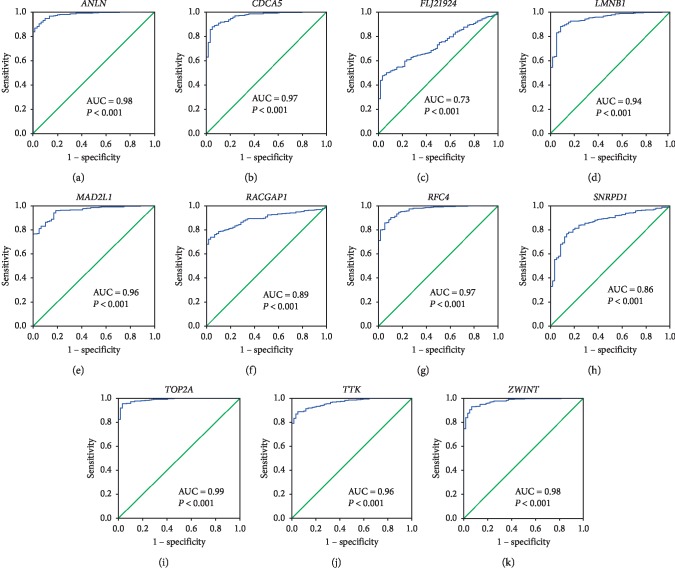
The ROC curves of 11 hub genes. These ROC curves described the diagnostic efficiency of the mRNA levels of (a) *ANLN*, (b) *CDCA5*, (c) *FLJ21924*, (d) *LMNB1*, (e) *MAD2L1*, (f) *RACGAP1*, (g) *RFC4*, (h) *SNRPD1*, (i) *TOP2A*, (j) *TTK*, and (k) *ZWINT* for LUADs and normal tissues.

**Figure 12:**
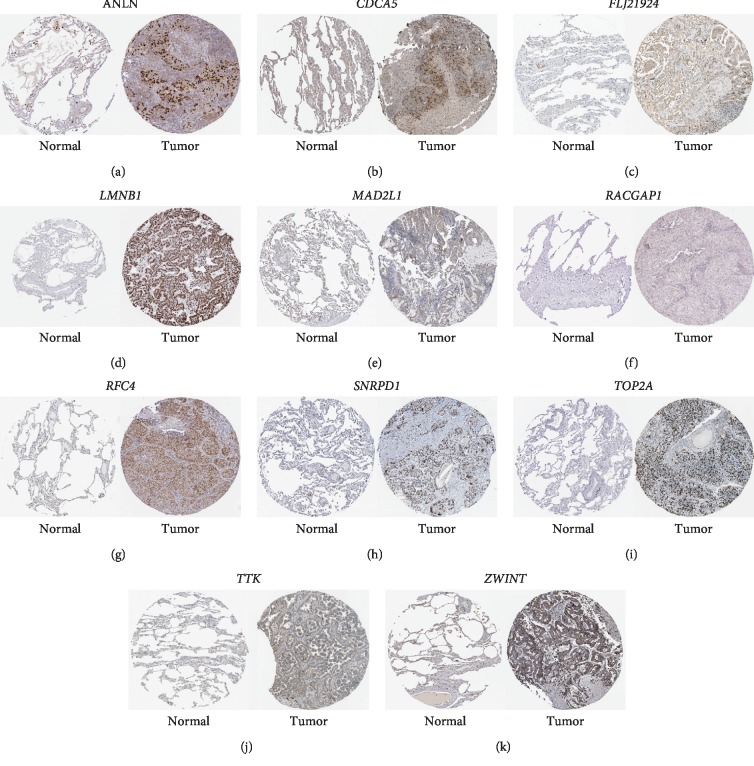
The results of the immunohistochemistry staining of the 11 hub genes obtained from the Human Protein Atlas database. Representative images of immunohistochemistry staining of (a) *ANLN*, (b) *CDCA5*, (c) *FLJ21924*, (d) *LMNB1*, (e) *MAD2L1*, (f) *RACGAP1*, (g) *RFC4*, (h) *SNRPD1*, (i) *TOP2A*, (j) *TTK*, and (k) *ZWINT* in normal (left) and LUADs (right). These images were obtained from the Human Protein Atlas database and the corresponding web links were given in supplementary [Supplementary-material supplementary-material-1].

Under the condition of setting cut-off value as |cor.geneModuleMembership| ≥ 0.8 and |cor.geneTraitSignificance| ≥ 0.2, 29 genes in the brown module were identified as hub genes. Among these 29 genes, we found that the expression levels of 11 genes were significantly related with worse overall survival (OS) (Figure 8) and progression-free survival (PFS) (Figure 9), which included *ANLN*, *CDCA5*, *FLJ21924* (also known as *QSER1*), *LMNB1*, *MAD2L1*, *RACGAP1*, *RFC4*, *SNRPD1*, *TOP2A*, *TTK*, and *ZWINT*. In addition, the data of TCGA showed that the mRNA levels of the panel of genes were significantly (all *P* values <0.0001) upregulated in primary LUAD tissues compared with normal tissues (Figure 10). Notably, ROC curves showed that the whole 11 identified genes had highly diagnostic efficiencies to distinguish tumors from normal tissues (Figure 11). The results of immunohistochemical staining in the Human Protein Atlas database indicated that the protein abundances of *ANLN*, *CDCA5*, *FLJ21924*, *LMNB1*, *MAD2L1*, *RACGAP1*, *RFC4*, *SNRPD1*, *TOP2A*, *TTK*, and *ZWINT* were higher in LUAD tissues than normal lung tissues (Figure 12).

## 4. Discussion

Within the last decade, the finding of *EGFR* mutation and *ALK* rearrangement in LUAD patients have propelled the development and application of targeted therapies including EGFR tyrosine kinase inhibitors (TKIs) and crizotinib. Actually, targeted therapies have been the standard care for advanced LUAD patients harboring *EGFR* or *ALK* alterations. Following the implementation of large-scale genomic studies of Clinical Lung Cancer Genome Project, it has been realized that LUADs are more likely to be driven by single somatic alteration than squamous cell carcinomas [19]. Therefore, LUADs could more easily benefit from various molecular targeted therapies. Despite the huge clinical benefits brought by targeted therapies in multiple subtypes of patients, the 5 years survival rate of lung cancer patients is still less than 20% [20]. More alterations related to LUAD are continually discovered. For example, Keap1/Nrf2 and Dach1/Eya/Six signaling pathways are involved in the oncogenesis and therapeutic resistance of NSCLC [21–23]. It is generally believed that due to the high heterogeneity in genome and complex mutation spectrum, genetic alteration-guided molecular targeted therapy has a long way to go. A more comprehensive understanding of the genetic traits of tumors is the foundation of personalized medicine for LUAD. In this study, we analyzed gene expression data from GEO and TCGA databases to identify biomarkers heralding tumorigenesis and the poor outcomes of LUADs.

This WGCNA was performed based on GSE11969 which was aiming to explore the coexpression modules related to clinical outcomes of LUAD patients. To save computer memory and data processing time, we selected top 50% most differentially expressed genes to generate a coexpression network. Eventually, we found the brown modules were significantly correlated with patients' clinical traits and identified 29 hub genes. After validating in multiple public databases, we noticed the elevated mRNA levels of 11 genes strongly indicated the poorer PFS and OS of LUADs. The preliminary results showed that this panel of genes including *ANLN*, *CDCA5*, *FLJ21924*, *LMNB1*, *MAD2L1*, *RACGAP1*, *RFC4*, *SNRPD1*, *TOP2A*, *TTK*, and *ZWINT* were potential adverse prognostic factors and tumorigenesis biomarkers for LUAD patients.

Human *ANLN* is a homologue of anillin (an actin-binding protein in *Drosophila*) [24]. *ANLN* is a cell cycle-associated protein which not only participates in cytokinesis but also promotes the growth and migration activities by PI3K-Akt and Rho signaling pathways [24, 25]. Previous studies demonstrated that the high protein abundance of *ANLN* was the predictive biomarker for multiple cancers such as breast cancer [26], lung squamous cell carcinoma [27], and prostate cancer [28]. Our results showed that upregulated *ANLN* was related to shortened PFS and OS of LUADs. However, the exact mechanisms by which *ANLN* induces LUAD progression and affects patients' outcomes are still unclear. Human *CDCA5* (also known as Sororin) is the core regulator of the cohesion of sister chromatins and the removal of cohesion [29]. Up to now, there is no direct evidence that *CDCA5* relates to the initiation and development of any subtype of lung cancer. However, the role of *CDCA5* has been confirmed in other cancers including hepatocellular cancer [30], breast cancer [31], gastric cancer [32], and colorectal cancer [33]. Accumulating studies showed that increased *CDCA5* level was a diagnostic biomarker and risk factor for numerous cancers which could enhance the tumor cells' capability of proliferation and metastasis by oncogenic ERK5-AP-1 pathway [33, 34].

*LMNB1* (also termed as lamin B1) is the vital component of nuclear structure which locates between inner nuclear membrane and peripheral heterochromatin [35]. *LMNB1* and its binding protein form the nuclear matrix and modulate a number of biology functions such as genome replication, DNA damage repair, transcription, as well as nuclear stability [35]. Moreover, *LMNB1* is regarded as a cancer-associated protein. It was reported that the expression of *LMNB1* positively correlated with low-grade differentiation and the risk of distant metastasis [36]. In addition, the silencing of *LMNB1* impaired tumorigenicity, tumor invasion, and cell proliferation in pancreatic cancer cells [36]. Additionally, the overexpression of *LMNB1* was the biomarker indicating the occurrence of retinoblastomas and poor prognosis of colon cancers [35, 37]. The relationship between *LMNB1* and LUAD has not been observed previously and *LMNB1* might be a promising predictive biomarker and molecular target.

*RACGAP1* (also referred to as Rac GTPase activating protein 1) is a cytokinesis-regulatory protein which is often overexpressed in multiple cancers. Knocking out *RACGAP1* in hepatocellular carcinoma cells hampered cytokinesis and induced cell apoptosis [38]. Upregulated *RACGAP1* predicted the poor outcomes in patients with hepatocellular carcinoma [38], ovarian cancer [39], and bladder cancer [40]. However, there are no preclinical or clinical studies demonstrating the role of *RACGAP1* in LUAD. Similar to *RACGAP1*, *RFC4* (also known as human replication factor C4) participates in the regulation of cell cycle as well, which acts as a clamp loader in DNA replication. Previous studies showed that the high expression of *RFC4* was the biomarker of tumorigenesis, poor survival [41], as well as chemotherapy resistance of colorectal cancer patients [42]. Given no experimental results supporting the role of *RFC4* in LUAD, further investigation is needed.

There are rare studies investigating the role of *FLJ21924* (QSER1) and *SNRPD1* in cancers. On the contrary, the predictive roles of *MAD2L1* and *ZWINT* for LUADs have been reported [43, 44]. Moreover, *TOP2A* (DNA topoisomerase II alpha) encodes a DNA topoisomerase which is a well-studied cancer-associated protein [45]. Several anticancer agents targeting DNA topoisomerase have been applied in clinical practice [46]. Apart from *TOP2A*, agents targeting another oncogenic molecular *TTK* are under development [47].

WGCNA is a widely adopted method to perform large-scale data mining. It is generally believed that genes in the same module share similar biology function. In our study, we found that the genes in the clinical significant module were significantly enriched in cell cycle and DNA damage repair-related pathways. However, the results might be misinterpreted due to the tissue contaminations or other technical defaults. To make our results more stable and decrease the potential biases, we conducted a comprehensive validation in other databases including TCGA and the Human Protein Atlas.

## 5. Conclusion

In conclusion, based on the WGCNA data mining technique, our study identified a panel of tumorigenesis and poor prognosis-related biomarkers. Among the screened hub genes, the mRNA levels of *ANLN*, *CDCA5*, *FLJ21924*, *LMNB1*, *MAD2L1*, *RACGAP1*, *RFC4*, *SNRPD1*, *TOP2A*, *TTK*, and *ZWINT* significantly related to worse survival data of LUAD patients. Our results indicated that these 11 genes might be potential predictive biomarkers and molecular targets in LUAD treatment.

## Data Availability

Previously reported gene expression data (GSE11969) were used to support this study and are available at the NCBI-GEO database (https://www.ncbi.nlm.nih.gov/geo). Pretreated TCGA data were obtained from https://xena.ucsc.edu/.

## Supplementary Materials

Supplementary Materials Supplementary file 1: the correlation analysis of 9 modules. Supplementary File 2: for the given data of patients' differentiation grade, the correlation analysis was based on the results of gene significance and cor.geneModuleMembership. Supplementary File 3: the web links of images from the Human Protein Atlas database in the paper.
