# Supplementary material for: Identifying Tumorigenesis and Prognosis-Related Genes of Lung Adenocarcinoma: Based on Weighted Gene Coexpression Network Analysis
Source: Biomed Res Int. 2020 Feb 28;2020:4169691. doi: 10.1155/2020/4169691 (PMC7035528; doi:10.1155/2020/4169691)
Supplement: Supplementary Materials — Supplementary file 1: the correlation analysis of 9 modules. Supplementary File 2: for the given data of patients' differentiation grade, the correlation analysis was based on the results of gene significance and cor.geneModuleMembership. Supplementary File 3: the web links of images from the Human Protein Atlas database in the paper. [file 4169691.f1.pdf]

Supplementary file 1

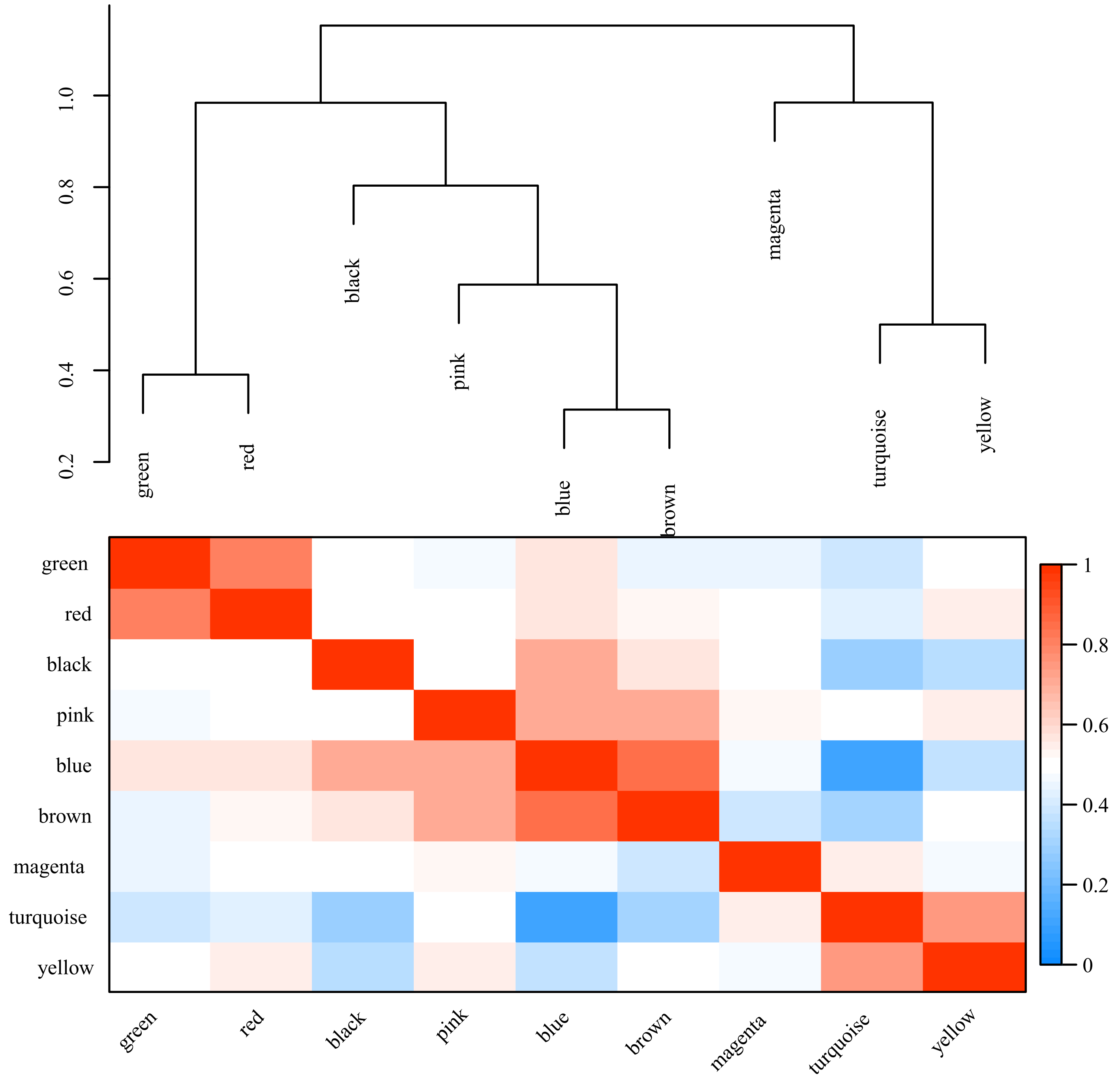

Supplementary file 2

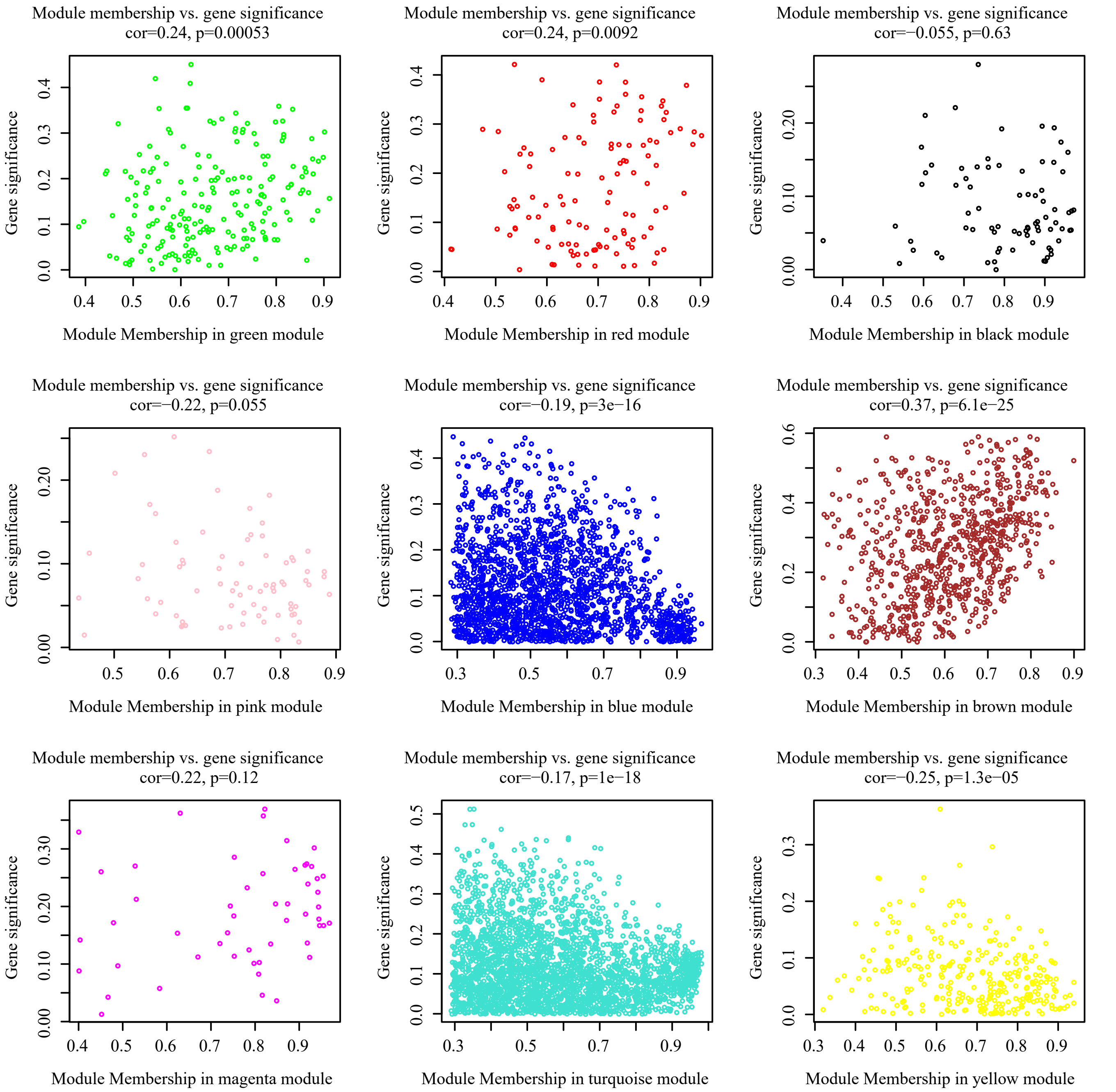

Supplementary file 3:

The web links of images from the Human Protein Atlas database in the paper:
ANLN in normal tissues: https://www.proteinatlas.org/ENSG00000011426-ANLN/tissue/lung#img;
ANLN in tumors: https://www.proteinatlas.org/ENSG00000011426-ANLN/pathology/tissue/lung+cancer#img;

CDCA5 in normal tissues: https://www.proteinatlas.org/ENSG00000146670-CDCA5/tissue/lung#img;
CDCA5 in tumors: https://www.proteinatlas.org/ENSG00000146670-CDCA5/pathology/tissue/lung+cancer#img;

FLJ21924 (also known as QSER1) in normal tissues: https://www.proteinatlas.org/ENSG00000060749-QSER1/tissue/lung#img,
FLJ21924 in tumors: https://www.proteinatlas.org/ENSG00000060749-QSER1/pathology/tissue/lung+cancer#img;

LMNB1 in normal tissues: https://www.proteinatlas.org/ENSG00000113368-LMNB1/tissue/lung#img;
LMNB1 in tumors: https://www.proteinatlas.org/ENSG00000113368-LMNB1/pathology/tissue/lung+cancer#img;

MAD2L1 in normal tissues: https://www.proteinatlas.org/ENSG00000164109-MAD2L1/tissue/lung#img;
MAD2L1 in tumors: https://www.proteinatlas.org/ENSG00000164109-MAD2L1/pathology/tissue/lung+cancer#img;

RACGAP1 in normal tissues: https://www.proteinatlas.org/ENSG00000161800-RACGAP1/tissue/lung#img;
RACGAP1 in tumors: https://www.proteinatlas.org/ENSG00000161800-RACGAP1/pathology/tissue/lung+cancer#img;

RFC4 in normal tissues: https://www.proteinatlas.org/ENSG00000163918-RFC4/tissue/lung#img;
RFC4 in tumors: https://www.proteinatlas.org/ENSG00000163918-RFC4/pathology/tissue/lung+cancer#img;

SNRPD1 in normal tissues: https://www.proteinatlas.org/ENSG00000167088-SNRPD1/tissue/lung#img;
SNRPD1 in tumors: https://www.proteinatlas.org/ENSG00000167088-SNRPD1/pathology/tissue/lung+cancer#img;

TOP2A in normal tissues: https://www.proteinatlas.org/ENSG00000131747-

TOP2A/tissue/lung#img;
TOP2A in tumors: https://www.proteinatlas.org/ENSG00000131747-TOP2A/pathology/tissue/lung+cancer#img;

TTK in normal tissues: https://www.proteinatlas.org/ENSG00000112742-TTK/tissue/lung#img;
TTK in tumors: https://www.proteinatlas.org/ENSG00000112742-TTK/pathology/tissue/lung+cancer#img;

ZWINT in normal tissues: https://www.proteinatlas.org/ENSG00000122952-ZWINT/tissue/lung#img;
ZWINT in tumors: https://www.proteinatlas.org/ENSG00000122952-ZWINT/pathology/tissue/lung+cancer#img;
